# A comparative evaluation of the sniffing, the simple head extension and the head hyperextension positions for laryngoscopic view and intubation difficulty in adults undergoing direct laryngoscopy

**DOI:** 10.1007/s00405-023-08386-y

**Published:** 2023-12-29

**Authors:** Y. H. Hadhoud, Mohamed A. Baraka, Mohamed Saleh, Ahmed M. Refaat

**Affiliations:** 1 Phoniatrics Unit, Otorhinolaryngology Department, Faculty of Medicine, Ain Shams University, Nasr City, Cairo, Egypt; 2 Assistant Professor of Anesthesia, Intensive Care and Pain Management, Faculty of Medicine, Ain Shams University, Cairo, Egypt

**Keywords:** Sniffing position, Suspension laryngoscopy, Simple extended position, Hyperextended position, Laryngeal view

## Abstract

**Objective:**

This work aimed to compare between the laryngoscopy positions; sniffing, simple head extension and head hyperextension positions to assess whether the laryngeal view, intubation time and intubation difficulty could improve with one of these positions than the others.

**Design:**

Prospective randomized three arms clinical trial.

**Setting:**

Operation room, the phoniatrics unit [removed for blind peer review].

**Participants:**

The study included 75 cases with 25 cases in each group. Group "A" with head in the sniffing position, Group "B" with the head in simple extension position, Group "C" with head in hyperextension position.

**Results:**

The three groups were compared regarding intubation time and laryngoscopic view time. Intubation time showed statistically significant difference between the three groups. Mean of sniffing group (No. = 25) was 13.19 s (± 3.35). Mean of simple extension group (No. = 25) was 11.29 s (± 3.14). Mean of Hyperextension group (No. = 25) was 14.39 s (± 4.14). Laryngoscopic view time showed statistically highly significant difference between the three groups. Mean of sniffing group (No. = 25) was 17.19 s (± 7.27). Mean of simple group (No. = 25) was 12.18 s (± 4.46). Mean of hyperextension group (No. = 25) was 17.08 s (± 6.51).

**Conclusion:**

Comparing the sniffing, the simple extension and the hyperextension positions, the simple extension position showed the best time regarding intubation time and laryngoscopic view time.

## Introduction

Direct laryngoscopy (DL) enables visualization of the larynx. It is used during general anesthesia, surgeries around the larynx and resuscitation measures [1]. Suspension DL, in which the larynx and its surroundings are visualized using a rigid laryngoscope, is the major technique used for laryngeal surgeries [2]. Visualizing the larynx facilitates endotracheal intubation. Successful endotracheal intubation during general anesthesia necessarily requires a line of sight to the larynx achieved by positioning the head and neck and retracting the tongue and soft tissues of the floor of the mouth by a laryngoscope [3].

Direct laryngoscopy is a dynamic process that should start with proper positioning of the patient’s head and neck for optimal laryngeal visualization. Inadequate positioning may result in poor laryngeal view and prolonged or failed attempts of tracheal intubation as a result of the inability to visualize the larynx [4]. Repeated tracheal intubation attempts may lead to patient morbidity. More than two laryngoscopic attempts can increase the incidence of airway and hemodynamic complications. Suboptimal laryngoscopy may lead to accidental esophageal intubation which can cause serious complications [5].

The common test used for preoperative airway evaluation is modified Mallampati test that can predict difficult intubation depending on the oral cavity structures [6].

**Fig. 1:**
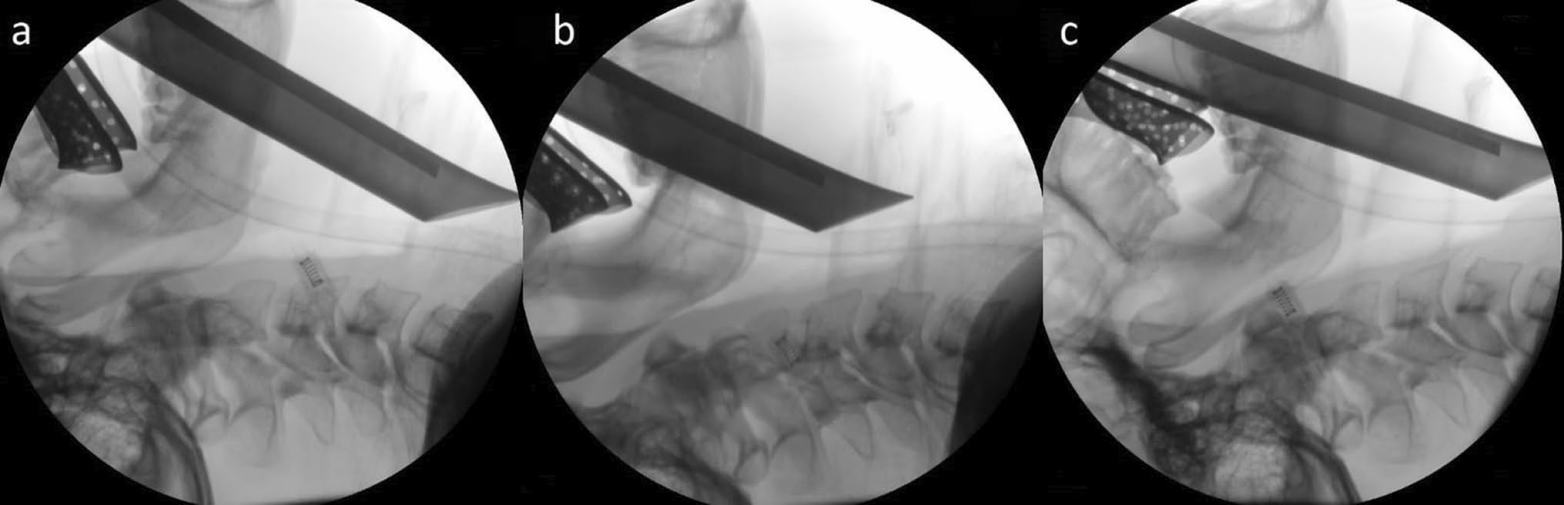
Intra-operative lateral X-rays of an informed anaesthetized patient, his head and neck were put in the three positions with the laryngoscope blade and the intubation tube inside

There are three head and neck positions proposed to facilitate ventilation and visualization of the glottis for intubation; sniffing, simple head extension and head hyperextension positions (Fig. 1).

In sniffing position, the neck must be flexed on the chest; typically, by elevating the head with a 7 cm-high ring cushion placed under the occiput and extending the head on the atlanto-occipital joint [7].

In simple head extension position, the patient is lying flat and there is passive flexion of the lower neck and head extension with no head elevation [8].

In head hyperextension position, the patient is lying flat and a 7 cm-high cushion is placed horizontally under the shoulders with straightening of the neck and tilting the head back keeping the mouth and throat in a straight line [9].

The sniffing position has traditionally been considered the optimal position of the head and neck for successful direct laryngoscopy [10]. However, the superiority of the sniffing position for laryngoscopy has been questioned as it was demonstrated that the sniffing position does not achieve alignment of the axes of the mouth, pharynx and the larynx [11].

### Aim of the work

This work aimed to compare between the laryngoscopy positions; sniffing, simple head extension and head hyperextension positions to assess whether the laryngeal view, intubation time and intubation difficulty could improve with one of these positions than the others.

## Materials and methods

The study protocol was approved by Ain Shams institute of ethical committee of human research and all patients involved in the study provided consent. Patient privacy and confidentiality were protected.

### Study design

This study is a prospective randomized three arms clinical trial, including voice patients attending the phoniatrics unit [removed for blind peer review], scheduled for elective suspension laryngoscopy surgery under general anesthesia and following the stated selection criteria during the period between October 2021 to October 2022.

#### Inclusion criteria

- Patients of either gender with age ranging from 18 to 65 years.
- Patients with Modified Mallampati class I–II.
- American Society of Anesthesiologists (ASA) physical status classification I, II.

#### Exclusion criteria

- Patients with age below 18 years or above 65 years.
- Patients with expected difficult intubation based on the preoperative airway assessment (Modified Mallampati class III–IV).
- American Society of Anesthesiologists (ASA) physical status classification III, IV.
- Patients with Difficult intubation.
- Patients with obvious malformation of neck or face.
- Patients with an unstable cervical spine.
- Patients with large tumors of the larynx.

### Sampling method

- The patients were randomly divided into three groups.
- Randomization is done by a computer-generated random number table.
- Sample Size: 75 cases were included in this study with 25 cases in each group.

Pachisia et al. [7] compared two methods only with a cross-over design reported a large effect size comparing the mean IDS and CL grade. Assuming an effect size of 0.4, a total sample size of at least 75 cases distributed equally between the three intervention groups achieves a power of at least 0.80 to detect a statistically significance difference using one-way ANOVA *F* test with level of significance of 0.05.

1. Group "A" the Sniffing group included supine patients intubated with head in the sniffing position.
2. Group "B" the Simple extension group included supine patients intubated with the head in simple extension position.
3. Group "C" the Hyperextension group included supine patients intubated with head in hyperextension position.

### Study procedures

All patients were subjected to the following in the operating room:

- Pre-operative assessment and Modified Mallampati classification while the patient was in sitting position. The preoperative airway evaluation was performed by one experienced anesthesiologist involved in the study to avoid inter-observer variability.
- The patient lied supine with the head placed in one of the laryngoscopy positions.
- All patients underwent standard monitoring and were given assisted ventilation with 100% oxygen via face mask followed by laryngoscopy after two and half minutes of muscle relaxant.
- An appropriate-sized Macintosh blade was used during laryngoscopy.
- Intubation is performed with appropriate-sized endotracheal tube.
- Suspension direct laryngoscopy using KANTOR-BERCI video-laryngoscopes with attached HOPKINS^®^ Straight Forward Telescope 15°, diameter 4 mm. with attached suitable medical monitor to record the procedures. Procedures were performed by one trained laryngologist involved in the study to avoid inter-observer variability.
- Minimal head and neck changes were required including external laryngeal pressure.

### Parameters of the study

- Intubation time (defined as the time from the instant the Macintosh blade touched the patient until tracheal intubation and removal of the laryngoscope blade from the mouth) and time for optimum laryngeal view by the suspension laryngoscopy (defined as the time from the instant the laryngoscope blade touched the patient until viewing the anterior commissure) were recorded for each patient.
- The primary outcome: time for optimum laryngeal view by the suspension laryngoscopy without calculating: suction time, changing blades time or mounting the laryngoscope holder and chest support.
- The secondary outcomes: intubation time.

## Results

### The demographic data of the study

This study was conducted on 75 voice patients scheduled for elective suspension laryngoscopy surgery under general anesthesia divided into 3 groups each group consisted of 25 patients and their data were analyzed. The age ranged between 22 to 63 years (Mean ± SD = 38.97 ± 11.16), including 42 males (56.0%) and 33 females (44.0%), there were 48 patients (64.0%) with Mallampati I and 27 patients (36.0%) with Mallampati II **(**Table 1**)**. By comparison of the demographic data between the three groups, they show non-significant difference and they were comparable (Table 2).

**Table 1:** The demographic data of the study

	No. = 75
Age	
Mean ± SD	38.97 ± 11.16
Range	22–63
Gender	
Male	42 (56.0%)
Female	33 (44.0%)
Mallampati	
I	48 (64.0%)
II	27 (36.0%)

**Table 2:** The demographic data of the three groups with comparative analysis

	Sniffing group (A)^a^	Simple extension group (B)^a^	Hyperextension group (C)^a^	Test value	*P* value	Sig.
	No. = 25	No. = 25	No. = 25			
Age						
Mean ± SD	39.64 ± 11.11	38.80 ± 11.09	38.48 ± 11.70	0.070^b^	0.932	NS
Range	24–63	23–62	22–62			
Gender						
Male	16 (64.0%)	15 (60.0%)	11 (44.0%)	2.273	0.321	NS
Female	9 (36.0%)	10 (40.0%)	14 (56.0%)			
Mallampati						
I	19 (76.0%)	12 (48.0%)	17 (68.0%)	4.514	0.105	NS
II	6 (24.0%)	13 (52.0%)	8 (32.0%)			

*P* value > 0.05: Non-significant (NS)

^a^Chi-square test

^b^One Way ANOVA test

### The comparative analysis of results of the three groups (Table 3)

**Table 3:** Comparative analysis of the three positions regarding intubation time and laryngoscopic view time

	Sniffing group (A)	Simple extension group (B)	Hyperextension group (C)	Test value^a^	*P* value	Sig.
	No. = 25	No. = 25	No. = 25			
Intubations time (in s)						
Mean ± SD	13.19 ± 3.35	11.29 ± 3.14	14.39 ± 4.14	4.811	0.011	S
Range	7–18.8	6–18.3	7.3–22.6			
Laryngoscope view time (in s)						
Mean ± SD	17.19 ± 7.27	12.18 ± 4.46	17.08 ± 6.51	5.322	0.007	HS
Range	8.1–40.1	4–23.8	5.8–32.7			

*P* value < 0.05: Significant (S); *P* value < 0.01: highly significant (HS)

^a^One Way ANOVA test

**Fig. 2:**
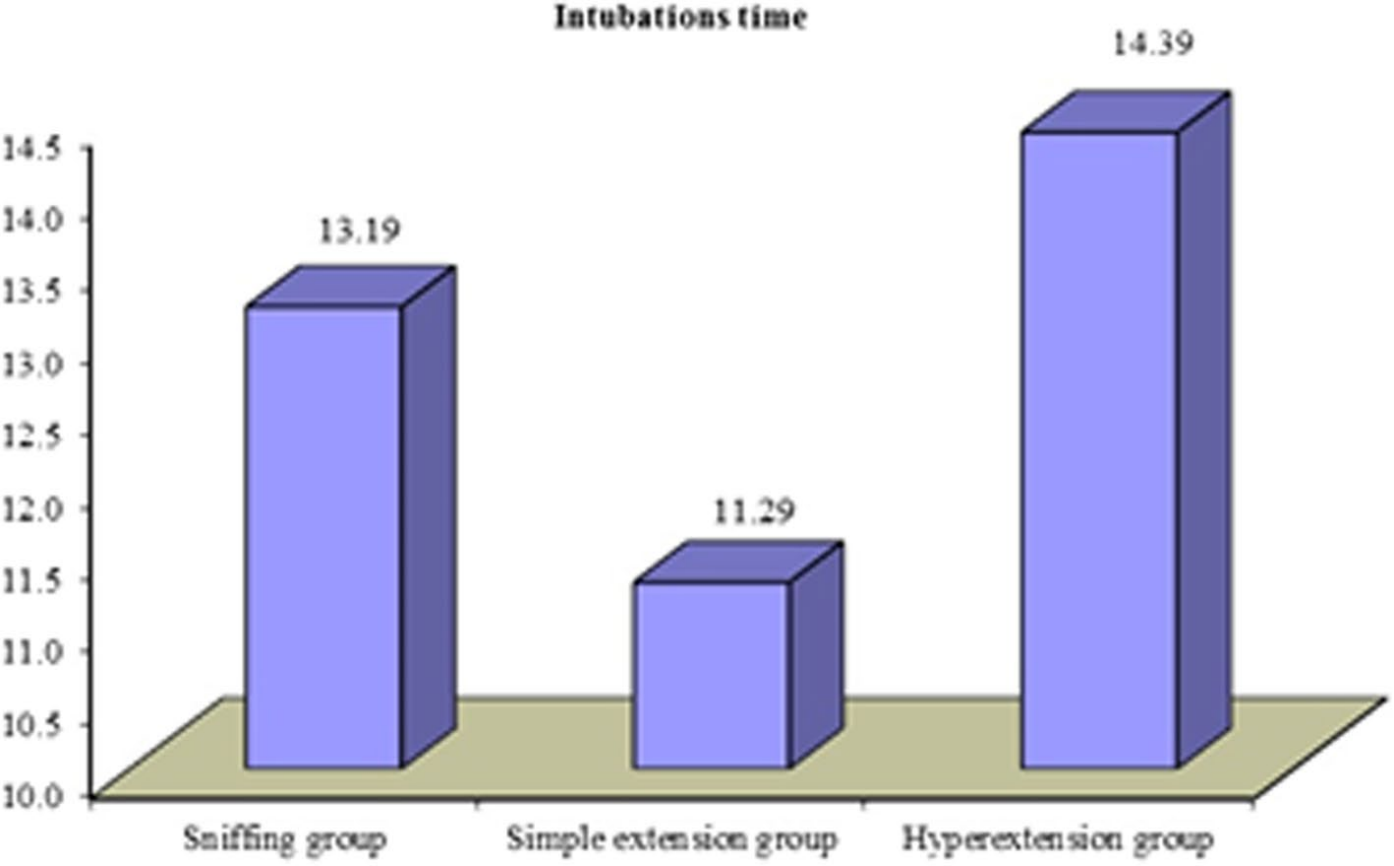
The distribution of the mean of intubation time among patients in the sniffing, the simple extension and the hyperextension groups. The figure shows that the mean of intubation time of the simple extension group (11.29) was approximately 20% lower than hyperextension group (13.19) and 10% lower than the sniffing group (14.39)

**Fig. 3:**
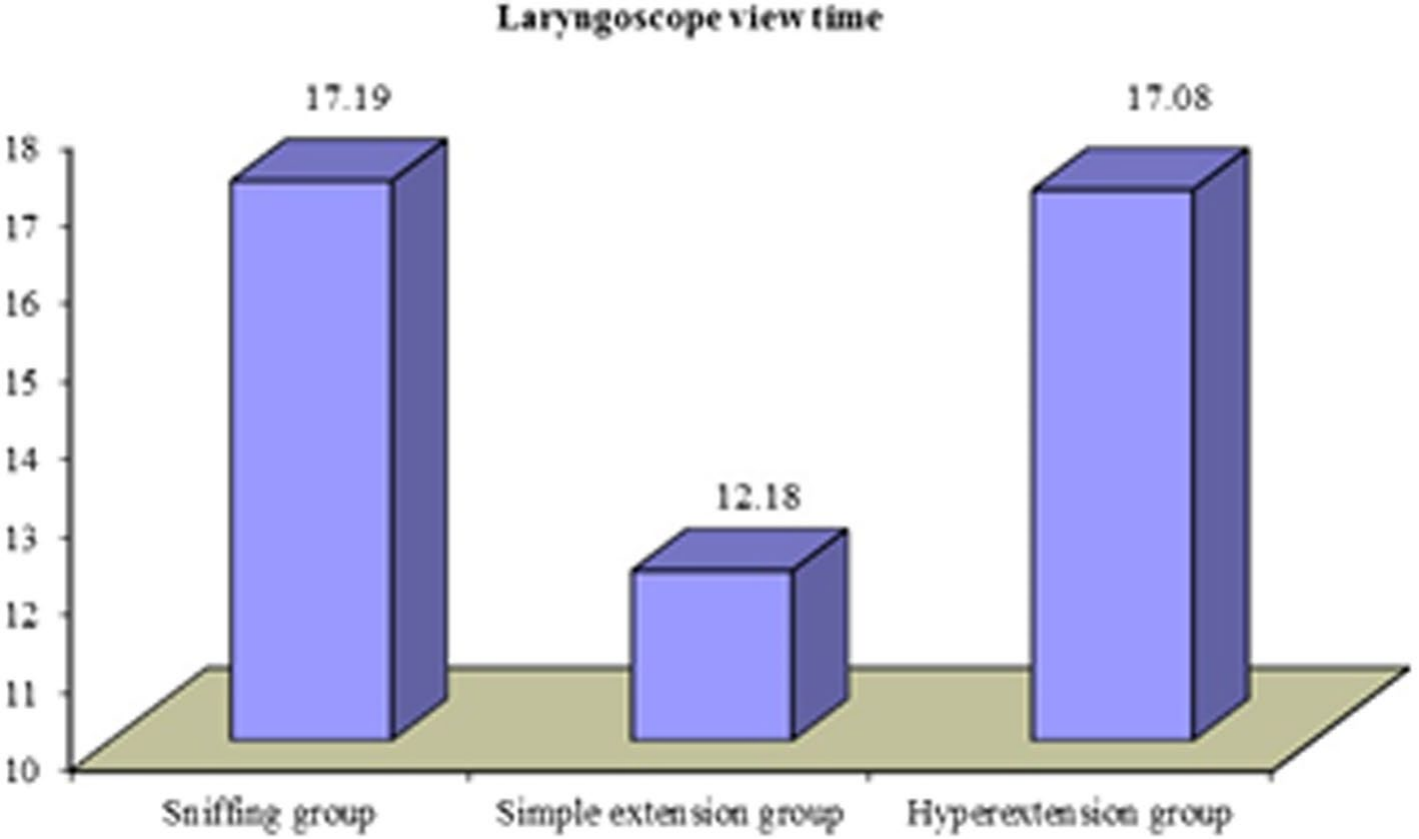
The distribution of the mean of laryngoscopic view time among patients in the sniffing, the simple extension and the hyperextension groups. The figure shows that the mean of laryngoscopic view time of the simple extension group (12.18) was approximately 30% lower than hyperextension group (17.08) and the sniffing group (17.19)

The three groups were compared regarding intubation time and laryngoscopic view time. Intubation time showed statistically significant difference between the three groups. Mean of sniffing group (No. = 25) was 13.19 (± 3.35). Mean of simple group (No. = 25) was 11.29 (± 3.14). Mean of hyperextension group (No. = 25) was 14.39 (± 4.14). Laryngoscopic view time showed statistically highly significant difference between the three groups. Mean of sniffing group (No. = 25) was 17.19 (± 7.27). Mean of simple group (No. = 25) was 12.18 (± 4.46). Mean of hyperextension group (No. = 25) was 17.08 (± 6.51) (Figs. 2, 3).

## Discussion

Optimal positioning of the patient is an essential factor for laryngoscopy and tracheal intubation. The sniffing position has historically been considered the best. The theoretical basis of this position is to allow the glottic visualization by aligning the oral, pharyngeal and tracheal axes through flexion of the lower cervical spine and extension of the atlanto-occipital joint. However recent literature has questioned the anatomical bases for this tradition.

The results of this study indicate that there is significant difference between the three positions in the intubation and laryngoscopic view times obtained during direct laryngoscopy with a Macintosh blade and suspension laryngoscopy respectively in anesthetized patients. Comparing the sniffing, the simple extension and the hyperextension positions, the simple extension position showed the best times.

Although the present study intubation times (maximum 13.19 s) and laryngoscopic view times (maximum 17.19 s) seem to be short insignificant times, yet these times are net without counting added time for changing blades, mounting the laryngoscope holder, chest support or suction.

Using straightforward cases with Mallampati 1 or 2 and including only single surgeon and anesthesiologist in this study were meant to decrease the variables in comparing between these positions.

Dasari et al. [11] after applying MRI on 20 awake volunteers comparing the three head and neck positions, stated that sniffing position produce the smallest angle between the laryngeal and tracheal axes reducing anterior impingement of the tube or bougie on the anterior wall of the subglottis or trachea for better glottic view. The laryngeal axis is defined as a line passing through the center of cricoid cartilage to the base of the epiglottis while the tracheal axis is defined as a line passing through the center of intrathoracic part of trachea to the center of cricoid cartilage.

While Gupta et al. [12] stated that when maxillo-pharyngeal angle is towards 100°, direct laryngoscopy could be performed easily and when the angle is less than 90°, it is difficult to visualize the larynx at direct laryngoscopy. Maxillo-pharyngeal angle is the angle between the maxillary axis (the line parallel to the hard palate) and the pharyngeal axis (the line passing through the anterior portion of the first (atlas) and second cervical vertebra). Normally the maxillo-pharyngeal angle is greater than 100° and it can be used preoperatively to expect difficult laryngeal exposure.

**Fig. 4:**
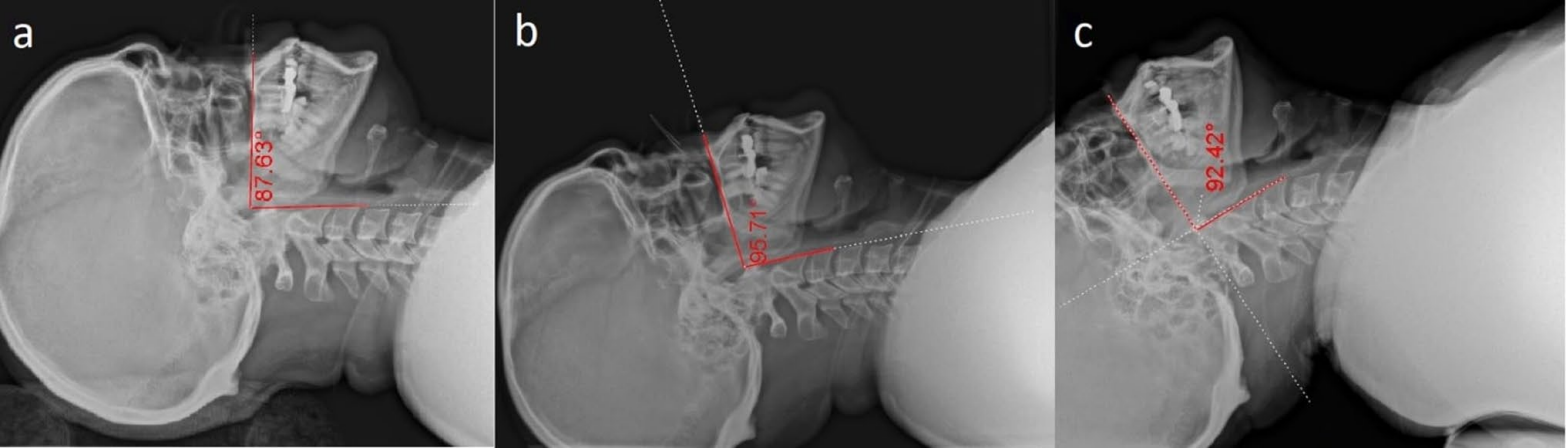
M–P angles in a non-anaesthetized volunteer with the head and neck been put in the three positions without using the laryngoscope blade

The Sniffing and head hyperextension positions showed maxillo-pharyngeal angles less than and near 90°, respectively while the simple head extension position showed angle towards 100° (Fig. 4).

Our results are comparable with Adnet et al. [13] who stated that systematic application of the sniffing position offered no appreciable advantage over simple head extension for improvement of glottic visualization with the use of direct laryngoscopy and a Macintosh blade. The sniffing position appears to be advantageous for obese and head extension-limited patients only.

Also comparable with Aziz et al. [14] in a secondary analysis from a comparative study who found that sniffing position was associated with higher risk of difficult glottic view when compared with simple extended “neutral” position.

A meta-analysis included six studies with 2759 participants showed that sniffing position did not improve glottic visualization or intubation time. In addition, the subgroup analysis comparing the sniffing position with the simple head extension position failed to show the superiority of the sniffing position and neither the intubation success rate nor intubation time differed significantly between the sniffing position and the other head positions [15].

**Fig. 5:**
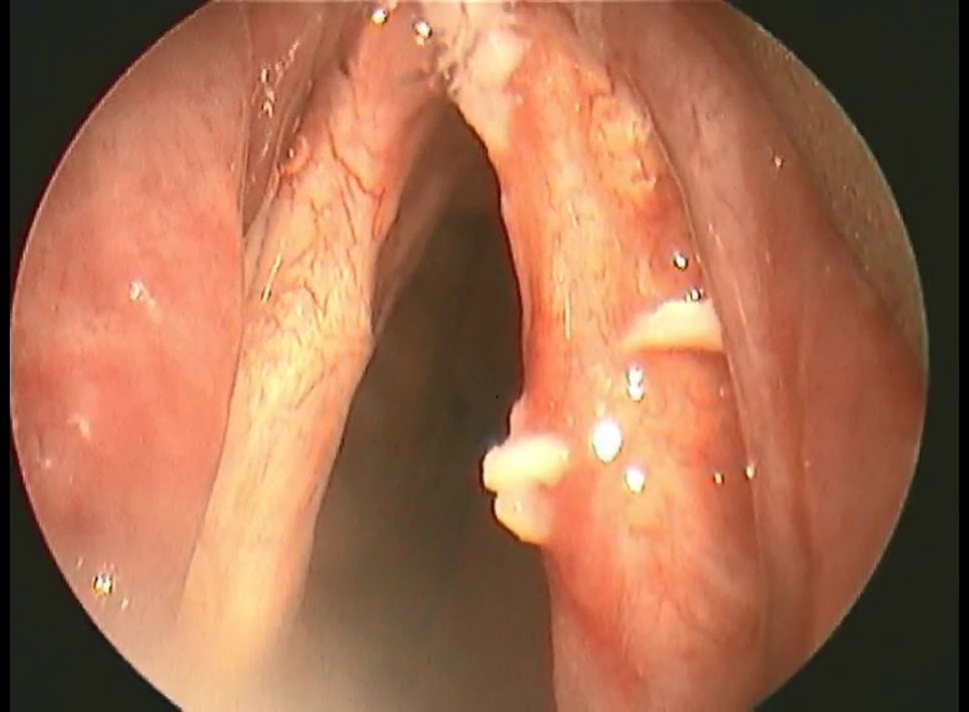
Laryngoscopic view showing anterior commissure exposed

On contrary, the rest of authors including Prakash et al. [16], Bhattarai et al. [17] and El-Orbany et al. [4] found that glottic visualization and intubation difficulty scores were better in sniffing position as compared to simple head extension and they concluded that the sniffing position should be used as a standard head position before attempts of direct laryngoscopy under general anesthesia (Fig. 5).

## Conclusion

This study presents some novel findings challenging the commonly accepted view that "sniffing" position provides the optimal access for laryngoscopy. Our finding are clinically relevant as the head hyperextension and sniffing positions involve cervical spine manipulation which can worsen plenty of functional and segmental restrictive cervical spine diseases such as fractures of cervical vertebrae, Acute whiplash, dislocation of the cervical vertebrae, Acute cervical disc herniation, recent cervical surgery, rheumatoid arthritis, osteoporosis, spondylosis, Down syndrome, Chiari malformations, cervical tumor/bony malignancy and vascular pathologies of the neck. While simple extension position is safe in all these functional and segmental restrictive cervical spine diseases as it doesn’t involve any cervical spine manipulation [17].

## Recommendations

This work is a starting point for other upcoming studies including patients with expected to difficult intubation and laryngoscopy view to assess how different head and neck positions and other factors such as BMI, neck movement, incisors distance and occlusion might affect intubation difficulty and laryngoscopic view [18].

Also, further studies including different surgeons and anesthesiologists should be considered to reduce bias if either of these positions are operator’s preference.

## Data Availability

The datasets used and/or analyzed during the current study are available from the corresponding author on reasonable request.
